# 

**Published:** 1899-08-05

**Authors:** 


					THE HOSPITAL. a The Standard Of highest purityr-Lancet. August 5th, 1899
CADBURY'S rf*> M CADBURY'S
(y COGOA j?> ^m MS~> COCOA
ABSOLUTELY FUR*. ^ NO DRUGS OR ALKALIES USED.
No. 671.m Vol. XXVI. [jXSJEJ Saturday,
August 5th, 1899.
r^Tm'] PRICE TWOPENCE.

				

